# Suicide in Italy: Epidemiological Trends, Contributing Factors, and the Forensic Pathologist’s Role in Prevention and Investigation

**DOI:** 10.3390/jcm14041186

**Published:** 2025-02-11

**Authors:** Saverio Gualtieri, Stefano Lombardo, Matteo Antonio Sacco, Maria Cristina Verrina, Alessandro Pasquale Tarallo, Angela Carbone, Andrea Costa, Isabella Aquila

**Affiliations:** Department of Medical and Surgical Sciences, Institute of Legal Medicine, University “Magna Graecia” of Catanzaro, 88100 Catanzaro, Italy; saverio.gualtieri@studenti.unicz.it (S.G.); stefano.lombardo@studenti.unicz.it (S.L.); matteoantoniosacco@gmail.com (M.A.S.); mariacristina.verrina@studenti.unicz.it (M.C.V.); alessandropasquale.tarallo@studenti.unicz.it (A.P.T.); angela.carbone1@studenti.unicz.it (A.C.); andrea.costa001@studenti.unicz.it (A.C.)

**Keywords:** suicide, prevention, psychiatry, autopsy, psychological autopsy

## Abstract

Suicide in Italy represents a serious public health problem, with significant data highlighting the urgency for prevention interventions. According to the epidemiological data, in the two-year period 2020–2021, 7422 suicides were recorded, representing an increase compared to previous years. Suicide is the most extreme self-harm. The contributing factors that surround this event are multiple, typically in conditions of serious distress or psychological distress, in particular in people suffering from serious psychiatric and/or mental disorders, such as depression. The role of the forensic pathologist in the context of suicide is crucial for ascertaining the contributing factors of death and for understanding the circumstances that lead to the suicidal act. Forensic medicine plays a crucial role in the analysis and understanding of suicides, addressing both the legal and medical implications. The aim of this study was to accurately describe the phenomenon of suicide in Italy. The accuracy of the review was very important in paying attention to the large difference in how the phenomenon manifests itself in the male population compared to the female population. The different ages at which suicide is committed were highlighted. The geographical difference between the North and the South and between the more urbanized areas compared to the rural areas where suicide is committed was analyzed. This scientific work also aimed to explore how forensic pathologists contribute to the resolution of complex forensic investigations. Psychological autopsy is an investigative method used primarily in cases of violent or questionable death, with the aim of understanding the psychological and social circumstances that led to an individual’s death. This practice is distinct from forensic autopsy, which focuses on the physical analysis of the body to determine the cause of death. The role of forensic pathologists in investigating suicide cases is crucial, as they not only determine the cause of death but also analyze the psychological implications that may have led to the extreme act. The main objective of a forensic pathologist in these cases is to gather and interpret evidence that can help understand the psychological and social context that influenced the decision to commit suicide, identifying any warning signs and underlying motivations and factors that may have contributed to the suicide. This approach provides valuable information for prevention, enhancing the understanding of the psychological mechanisms behind suicide and supporting targeted interventions in the future. The manuscripts also have an explanatory purpose and may have a therapeutic role in helping surviving relatives understand suicide. Knowledge of the messages contained in suicide notes could be useful for suicide prevention programs.

## 1. Understanding the Suicide: Analysis of Risk Factors

Suicide is a significant public health concern, with profound social and emotional consequences for individuals, families, and communities. In Italy, the phenomenon of suicide reflects a complex interaction of cultural, social, and economic factors, making it a critical area for investigation and intervention. Although Italy generally reports lower suicide rates compared to other European countries, notable regional and demographic disparities underscore the need for a more detailed understanding of the factors influencing this phenomenon [1,2,3,4,5].

Demographic trends also highlight significant disparities in suicide rates. Men are significantly more likely to die by suicide than women, a pattern influenced by factors such as economic stress, substance use, and access to lethal means [1,2,3,4,5]. Women, on the other hand, have higher rates of suicide attempts, which are often associated with untreated mental health conditions like depression and anxiety. Additionally, vulnerable populations, including the elderly, LGBTQ+ individuals, and migrants, face heightened risks due to social marginalization, discrimination, and barriers to accessing mental health care. Suicide is an act that involves the conscious decision to end one’s life, a complex reality that has its roots in numerous psychological, social, cultural, and economic factors. The term itself, deriving from the Latin “sui caedere”, or “to kill oneself”, reflects the tragedy of this phenomenon. The motivations that push an individual to commit this extreme act are many and can be influenced by a series of events and conditions ranging from psychological and psychiatric suffering to loneliness, from economic difficulties to traumas suffered, and from family conflicts to social pressure [1]. Suicide is often the epilogue of a long process of suffering, a painful journey that culminates in a definitive act that marks the end of a life. Understanding it requires an in-depth analysis of various factors that contribute to the genesis of such an act, including psychiatric disorders, socio-economic difficulties, and traumatic experiences [2,3]. One of the most common contributing factors of suicide is the presence of psychiatric and mental disorders, with depression emerging as one of the most relevant factors. Depression, in fact, can lead to a deep sense of despair and helplessness, which, in the long term, can push an individual to think that the only way out is death. Other psychiatric disorders, such as bipolar disorder, schizophrenia, and post-traumatic stress disorder, are also strongly associated with suicide [4,5]. In these cases, suicide can be seen as a solution to psychological suffering that seems unbearable and irreversible. However, it is important to note that not all those who suffer from psychiatric disorders attempt or commit suicide. In many cases, therapeutic and family support can prevent the development of suicidal thoughts [6,7]. In addition to psychological disorders, there are also external factors that can contribute to suicide. Situations of serious economic difficulty, prolonged unemployment, professional dissatisfaction, family or relationship crises, and social isolation can be triggers or exacerbators [8,9]. Social and family pressures, such as failure to meet professional or personal expectations, inadequacy with external standards, or difficulty relating to others, can lead an individual to feel as if there is no escape from suffering. In addition, traumatic experiences, such as physical or psychological abuse, harassment, bullying, and, more recently, cyberbullying, can have devastating effects on an individual’s psyche, contributing to the growth of suicidal thoughts. Another significant aspect that contributes to suicide is impaired self-esteem [10]. The feeling of the non-acceptance of oneself, the inability to handle situations of embarrassment or failure, and the fear of not living up to the expectations of oneself or others are all factors that can undermine an individual’s mental stability. People who are unable to adapt to life’s challenges or who see themselves as incapable of coping with them may develop suicidal thoughts as a means of ending their suffering.

However, it is important to note that not all those who suffer from psychiatric disorders attempt or commit suicide. In many cases, therapeutic and family support can prevent the development of suicidal thoughts [6,7]. In addition to psychological disorders, there are also external factors that can contribute to suicide. Situations of serious economic difficulty, prolonged unemployment, professional dissatisfaction, family or relationship crises, and social isolation can be triggers or exacerbators [8,9]. Social and family pressures, such as failure to meet professional or personal expectations, inadequacy with external standards, or difficulty relating to others, can lead an individual to feel as if there is no escape from suffering. In addition, traumatic experiences, such as physical or psychological abuse, harassment, bullying, and, more recently, cyberbullying, can have devastating effects on an individual’s psyche, contributing to the growth of suicidal thoughts. Another significant aspect that contributes to suicide is impaired self-esteem [10]. The feeling of the non-acceptance of oneself, the inability to handle situations of embarrassment or failure, and the fear of not living up to the expectations of oneself or others are all factors that can undermine an individual’s mental stability. People who are unable to adapt to life’s challenges or who see themselves as incapable of coping with them may develop suicidal thoughts as a means of ending their suffering. Substance use disorders significantly influence the risk of suicide by exacerbating psychological distress, impairing judgment, and increasing impulsivity [11]. Alcohol and drug abuse are often implicated in suicide cases, as they can intensify feelings of hopelessness and despair [11]. Forensic investigations should therefore include a thorough evaluation of substance use history, as it provides critical context for understanding the deceased’s state of mind and contributing factors leading to their death. Additionally, Émile Durkheim’s seminal work on suicide highlights the social dimensions of the phenomenon, emphasizing how varying degrees of social integration and regulation can influence suicidal behavior [12]. Durkheim’s work on suicide, particularly his book Le Suicide published in 1897, is considered foundational in understanding the social dimensions of this phenomenon [12]. Durkheim approached suicide not as merely an individual act but as deeply influenced by societal factors. He argued that the structure and dynamics of society play a critical role in shaping the conditions that lead to suicide, emphasizing the importance of social integration and regulation. According to Durkheim, individuals who feel disconnected from their social groups or experience a lack of belonging are at a higher risk of suicide. This sense of isolation can lead to feelings of purposelessness and detachment, which are often precursors to suicidal behavior. Durkheim also explored how excessive societal pressures or rigid norms could contribute to suicidal tendencies. For example, in societies where individuals feel overly controlled or unable to exercise personal freedom, the stress of living under such conditions can manifest in self-destructive behaviors [12]. His analysis extended to the broader societal conditions, such as economic crises or political upheavals, which can disrupt the social fabric and leave individuals vulnerable. He was particularly interested in the way societal changes or breakdowns in traditional structures, such as family or religious institutions, could leave individuals feeling unsupported and adrift.

## 2. The Suicidal Process: From Ideation to Action

Suicide is rarely a sudden act [13]. Rather, it is the culmination of a long and gradual suicidal process, which develops over a period of time, during which the individual goes through different emotional and psychological stages [14]. This process usually begins with the perception of intolerable pain and growing despair in the face of life’s difficulties. At this stage, the person may experience a feeling of helplessness and disillusionment, believing that there are no solutions to their problems [15]. Emotions of sadness, loneliness, and frustration intensify, and the person may begin to develop suicidal thoughts. Suicidal ideation is the first step in this process [16]. These are thoughts, wishes, or worries that revolve around the idea of suicide but are not necessarily accompanied by an intention to act immediately. The individual may begin to consider suicide as a way to escape from their suffering but has not yet taken concrete action to carry it out [17]. These thoughts may oscillate between the desire to die and the desire to end the suffering without actually wanting to die. In some cases, the individual may also try to hide these thoughts from others, trying to deal with them alone, or, on the contrary, they may express them to friends or family members without taking any concrete action. The next, more dangerous phase is that of suicide attempts [18]. In this phase, the individual no longer simply thinks about suicide but takes action directly toward their goal. Suicide attempts are often a cry for help, a way to draw attention to their suffering. There are high- and low-lethality suicide attempts: some are extremely serious and close to death, while others are less lethal but equally dangerous to health. High-lethality suicide attempts are those in which the individual uses extremely dangerous methods, such as the use of firearms or suicide by hanging [19]. Low-lethality suicide attempts, on the other hand, may include the ingestion of small amounts of toxic substances or the attempt to harm oneself in a non-fatal way. The suicidal process, unfortunately, is not always linear. In many cases, people who have attempted suicide repeat the act multiple times. Multiple suicide attempts are a warning sign, as those who attempt them are significantly more likely to complete suicide in the future [20]. People who have attempted suicide more than once are also often more vulnerable to psychiatric and psychological disorders and may have a family history of suicide or emotional abuse, which further worsens their situation.

## 3. Material and Methods

This study combined a narrative review of the scientific literature using a rigorous methodology that involved several specialized sources of information. In particular, PubMed search engines, the “Istituto Superiore di Sanità” website, and the Istat databases were used. The analysis took into account a wide range of articles, publications, and reports that dealt with issues related to suicide in Italy, with the aim of providing a comprehensive and in-depth view of the phenomenon, its prevalence, and the factors that influence its onset. In order to conduct the search in a targeted and focused way, specific keywords were used, including “suicide and Italy”, “suicide notes”, and papers about the role of the forensic pathologist in suicides. The choice of these keywords was based on a particular interest in three main areas: first, the phenomenon of suicide in Italy, to understand the national dynamics and socio-cultural variables related to it; secondly, the study of “suicide notes” or notes left by suicides, which often represent one of the few traces that victims leave behind and which can offer crucial clues about their psychological state and the motivations behind the extreme gesture; and thirdly, the analysis of the role of the coroner, a fundamental figure in the process of investigating suicides, who contributes to providing a scientific and legal assessment of the event. This combination of sources and keywords allowed us to obtain a more complete and multifaceted overview of the phenomenon of suicide from a medico-legal, psychological, and sociocultural point of view and to identify possible areas of intervention to prevent this social drama.

## 4. Epidemiology of Suicide: A Global and Local Perspective in Italy

**Figure 1:**
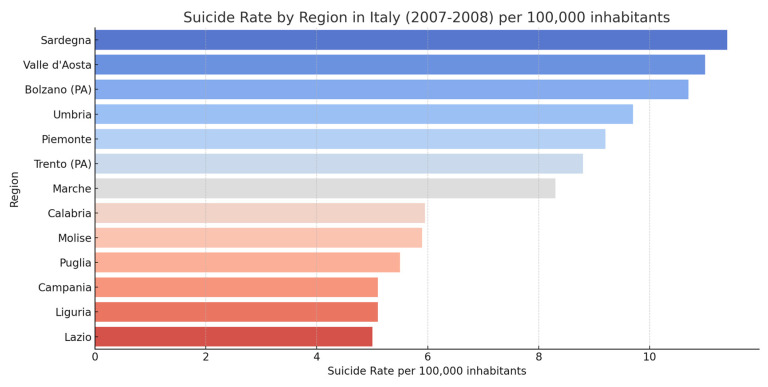
Graphics about suicide rates in Italy (2007–2008).

Suicide is a major public health problem globally. With over 800,000 deaths each year, suicide is a leading cause of death, and it is estimated that approximately one person dies by suicide every 40 s [21]. Suicide rates are particularly high among older people, but it is among young people that suicide takes on a tragic importance, ranking as the third leading cause of death among young people between 15 and 29, after road accidents and cancer. The data also show a worrying gender difference: men are much more susceptible to suicide than women. In the United States, in 2017, the male suicide rate was 3.67 times higher than the female suicide rate [22,23]. A similar trend has been observed in Italy, where male suicide rates are consistently higher than female suicide rates. For example, in 2018, 76.8% of suicides in Italy were men. However, the incidence of suicide is not uniform across the world [21,22,23,24,25,26]. Some countries, particularly those with high unemployment rates or severe socioeconomic difficulties, have significantly higher suicide rates. In Italy, for example, suicide rates are higher in Northern Italy, particularly in the Northeast regions [27]. This territorial variation is a demonstration of the fact that suicide is influenced not only by individual factors but also by socioeconomic, cultural, and political factors. Differences in suicide rates between Italian regions can be attributed to a combination of factors, such as the availability of health services, economic disparities, and the level of social support. Suicide is often influenced by external factors, such as economic crises or catastrophic events, which can aggravate the psychological condition of already vulnerable individuals. An example of this is the 2008 economic crisis, which saw an increase in suicide rates in Italy, especially among men in the central age groups (between 25–30 years and 65–69 years) [28]. Job loss, economic hardship, and fear of the future were determining factors in this critical period. Similarly, the SARS-CoV-2 pandemic has raised concerns about a possible increase in suicides due to social isolation, physical distancing, fear, and economic stress. However, data from Italy show that, despite initial predictions, suicide rates in 2020 did not increase dramatically compared to previous years [29]. Although there was a slight increase among older men and some women, overall suicide mortality decreased, partly reflecting support for containment measures and increased awareness of the psychological risks related to the pandemic. Regional variations in suicide rates are indeed influenced by a range of socioeconomic, cultural, and political factors. For example, the northern regions of Italy, such as Trentino-Alto Adige and Lombardy, have higher suicide rates compared to southern regions like Calabria and Sicily [30]. These differences can be attributed to various factors. Socioeconomically, the northern regions, while more developed, experience higher unemployment rates and job insecurity, which can contribute to greater psychological stress. Moreover, social isolation and loneliness, phenomena more common in rural or mountainous areas of the North, are also risk factors for suicide. Culturally, the South of Italy has stronger community traditions, which may foster a social support network that in part protects individuals from suicidal behavior. However, the stigma associated with mental illness in certain areas may limit help-seeking, contributing to higher suicide rates among those who suffer in silence. Politically, the management of healthcare resources and the presence of mental health policies vary across regions. Northern regions, generally wealthier, can afford to provide more accessible and structured mental health services, while some southern areas suffer from a shortage of resources and limited access to psychological and psychiatric treatments, thus increasing the risk of suicide [31]. These examples demonstrate how socioeconomic, cultural, and political factors have a significant impact on the epidemiology of suicide in Italy, highlighting the importance of a regional and context-specific approach in the prevention and treatment of suicidal behaviors (Figure 1) (Table 1).

## 5. The Role of Forensic Medicine: A Multidimensional Perspective and Italian Data

### 5.1. The Importance of Forensic Medicine in Suicide Analysis

Forensic medicine plays a fundamental role in understanding the circumstances of suicides and improving prevention strategies. Through the analysis of autopsies and judicial inspections, forensic pathologists can collect crucial evidence regarding the manner and circumstances that led to suicide [27,28]. Forensic medicine can also evaluate manuscripts, farewell letters, or any other type of communication left by the deceased person, which can provide further clues about their psychological state and the reasons that led to such an act. Furthermore, collaboration between forensic medicine and psychiatry is essential to addressing the complexity of suicide. An integrated analysis of the psychological condition of the victim and the social and economic circumstances that may have contributed to suicide allows for the development of more effective approaches to prevention. The synergy between psychiatrists, psychologists, forensic pathologists, and social workers is crucial to create timely and targeted interventions, especially in people at risk of suicide, such as those with a history of psychiatric disorders or who have been exposed to traumatic experiences. The analysis of the review highlighted the great difference in how the suicide phenomenon manifests itself in the male population compared to the female population.

### 5.2. Evaluation of Suicide Statistics in Italy

In Italy, according to ISTAT data from the “Survey on causes of death”, in 2018, 3699 people took their own lives (excluding foreigners and non-residents), of which 76.8% were men [29,30,31]. The suicide rate for men is 11.21 per 100,000 inhabitants, while for women it is 3.15 per 100,000, with a gender ratio (men/women) that has increased over time, increasing from 2.1 in 1980 to 3 in 2018 [29,30,31]. The review showed that suicide rates are higher in Northern Italy, particularly in the Northeast regions [10]. The analysis of age-specific rates for the year 2016 showed that, for men, the rate increased constantly, reaching a value of almost 20 cases per 100,000 inhabitants among the elderly over 70 years of age. For women, rates also increased with age, reaching a maximum of over 4 cases per 100,000 among women over seventy [29,30,31]. The frequency tends to undergo significant variations in certain eras. An example was the economic and financial crisis of 2008, which in Italy resulted in an increase in suicides among men in the central age groups (between 25–30 years and 65–69 years), while for women the variation was more contained [32]. The SARS-CoV-2 pandemic behaved differently [33,34,35]. Since the initial phase, great concern has emerged about the possible increase in suicide rates in the population. Lockdown, restrictions, fear, prolonged isolation, social distancing, and economic stress were thought to be important growth factors, but this was not the case. In Italy, the number of deaths by suicide in 2020 was 3712 (79% males, 21% females), demonstrating a reduction in both males (−2.8%) and females (−7.7%) compared to 2015–2019 [36]. Suicide rates decreased in most age groups of the population; an increase in mortality by suicide was found among men aged ≥75 years, among women aged ≥85 years, and to a lesser extent among women aged 45–54 years, but in all cases the increase was not statistically significant [36]. The 2017 ISTAT report also presented for the first time a descriptive analysis of the comorbidities associated with suicide. The aim was to provide a quantitative and qualitative measure of suicides, investigating the presence of a major illness known to the subject (physical or mental), which could have influenced the choice to commit suicide [30,31,32,33,34,35,36]. In all cases of suicide in the three-year period 2011–2013, 12,877 suicides were recorded (2812 women and 10,065 men), and approximately 1 in 5 suicide cases presented a significant associated morbidity (2401 deaths). The frequency of significant morbid states was higher with increasing age, and, in women, the proportion of suicides with associated morbidity was 27%, while, in men, it was 16%. In 737 suicides, the presence of significant physical illnesses was certified, and among these, 288 also presented a certified mental illness (mainly depression). In 1664 cases, the presence of only mental illnesses was reported (mainly depression and anxiety). About half of the suicides occurred at home [30,31,32,33,34,35,36]. Thirty percent of suicides in the presence of physical illnesses occurred in nursing homes. Suicide rates among men were inversely proportional to population density; therefore, it is more common in rural areas and conversely in urban areas for women, probably because men are more vulnerable to adverse social and economic factors associated with a lower population density. Men tended to use more lethal means, could conceive the suicide attempt more carefully, and more often carried it out [30,31,32,33,34,35,36]. Among males, the most frequently used methods to carry out suicide were hanging (52.2%), falling from high places (15.8%), and the use of firearms (13.7%). Among young people, this deadly choice was repeated among males aged 15–29, and hangings represented 42.7% of suicides followed by falling from a high place (24.8%); the third choice was being run over by a moving vehicle (13.8%), followed by the use of firearms (8.5%). Furthermore, for females, hanging (34.8%) and falling from high places (31.9%) were the two most frequently used methods, but in third place, it was drowning (8.0%), while the use of firearms was rare (2.1%) [30,31,32,33,34,35,36]. Therefore, forensic medicine also intersects with questions of biolaw, addressing the legal and moral needs related to life and death [33].

### 5.3. The Role of Linguistics

The study of linguistics plays a crucial role in understanding suicide notes, as it provides a window into the emotional and psychological state of individuals in their final moments [37]. By analyzing the language used in these notes, researchers can uncover patterns and themes that reflect the writer’s mental processes, emotional struggles, and social connections. For example, the choice of words, tone, and sentence structure often reveals feelings of despair, hopelessness, or even guilt. Words like “sorry”, “pain”, or “love” frequently appear, offering insights into how the individual perceives themselves and their relationships with others.

One of the most significant aspects of linguistic analysis in suicide notes is its ability to highlight cognitive distortions. Many notes express black-and-white thinking, such as using absolute terms like “always” or “never”, which indicate a sense of entrapment or finality. This rigid way of thinking often reflects the deep psychological pain that individuals experience and their perception that there is no alternative to ending their suffering.

Additionally, the tone of suicide notes varies widely [37]. Some are deeply apologetic, seeking forgiveness from loved ones, while others may be accusatory or reflective. The language used can also indicate whether the individual had a sense of closure or unresolved conflict. Notes written in a calm and organized tone may suggest a sense of resolution, while those filled with chaotic or fragmented thoughts often point to heightened emotional distress. Linguistic analysis also sheds light on the social connections of the individual. Many suicide notes are addressed to specific people, revealing the writer’s final attempts to communicate their feelings, whether to apologize, express love, or provide explanations. The language in these notes often reflects a complex mix of emotions, including a desire to alleviate the burden on loved ones, even if the act itself causes pain.

## 6. The Synergy Between Forensic Medicine and Psychiatry

A forensic pathologist is a professional who investigates unexpected or violent deaths, determining the cause and manner of death. In particular, in the case of suicides, the pathologist must establish whether the death was due to suicide, homicide, accident, or natural causes. This process involves an in-depth analysis of the deceased’s medical history, the circumstances of death, and, if necessary, the performance of autopsies to clarify any doubts [38,39,40]. Recent studies have highlighted that the risk of suicide is often not recognized in the medical field, despite many patients having had contact with doctors in the weeks preceding the suicide. It is essential to improve the diagnosis and treatment of psychiatric disorders to prevent such tragic events.

The training of coroners and their integration into mental health teams can contribute to the better identification and management of suicide risk. Forensic medicine offers a significant contribution to understanding and managing suicides through the analysis of deaths through the performance of judicial inspections, autopsies, and the evaluation of manuscripts often released by those who commit this extreme act. The synergy between forensic medicine and psychiatry has also emerged, which is crucial to address the complexity of suicide and improve prevention strategies [38,39,40]. For example, in one clinical hypothetical case, a forensic pathologist investigating a presumed suicide by hanging consulted with a psychiatrist to interpret the deceased’s psychological state. The psychiatrist’s analysis of the individual’s medical history and previous psychiatric evaluations revealed evidence of dissociative behaviors, leading to a reconsideration of the cause of death as accidental. This interdisciplinary collaboration demonstrates the importance of integrating psychiatric insights into forensic investigations to clarify ambiguous cases and prevent potential misclassifications. Another possible example involved the evaluation of a suicide note left by an individual who appeared to have died from self-inflicted injuries. The forensic pathologist’s examination of the physical evidence was complemented by the psychiatrist’s interpretation of linguistic and emotional cues within the note, which indicated significant untreated depression. This collaborative effort not only confirmed the cause of death but also provided critical information for public health strategies aimed at addressing gaps in mental health care.

The role of the medical examiner in the context of suicide is crucial in determining the cause of death and understanding the circumstances that led to the suicide [41,42,43]. The functions of the medical examiner in the case of death due to suicide can be divided into five phases: (1) Investigating the Cause of Death: The medical examiner is responsible for investigating deaths that occur under unclear circumstances, including suicides. They must determine whether the death occurred due to natural causes, accident, homicide, suicide, or other undetermined circumstances. This process involves a thorough analysis of the physical evidence and available testimonies; (2) Autopsy, Diagnostic Investigations, and Analysis: In the case of suicide, the medical examiner may perform an autopsy to gather vital information that can clarify the circumstances of the death. The autopsy helps to identify any mental illness or other conditions that may have influenced the behavior of the deceased; (3) Differential Diagnosis: It is essential for the medical examiner to distinguish between suicide, homicide, and accidental death. This requires careful assessment of the circumstances and evidence, such as the position of the body, the means used, and any notes left by the deceased; (4) Legal Aspects: Suicide has significant legal implications, especially in relation to liability and insurance issues [41,42,43]. For example, insurance policies may have specific clauses regarding suicide, and the coroner must be able to provide a clear and accurate assessment of the circumstances to support legal and insurance decisions; (5) Family and Community Support: In addition to investigations, the coroner may also play a role in suicide awareness and prevention, collaborating with other professionals to improve understanding of the warning signs and resources available to people in crisis. To enhance the synergy between forensic medicine and psychiatry, targeted training programs for professionals involved in suicide assessments are essential. Forensic pathologists, psychiatrists, and other mental health professionals should receive education in recognizing suicide risk factors, interpreting behavioral warning signs, and understanding the psychological context of suicide through methods such as psychological autopsies [41,42,43]. Training should also cover advanced forensic interviewing techniques and the analysis of suicide notes to uncover critical clues about the deceased’s mental state. Moreover, interdisciplinary workshops or case simulations can prepare teams to navigate complex scenarios collaboratively. Emphasis should also be placed on sociocultural considerations, as societal pressures and cultural norms often play a role in suicidal behavior. Additionally, professionals should be trained in providing support to bereaved families, equipping them with skills to communicate findings sensitively and guide loved ones through the investigative process.

To illustrate the collaborative roles of various professionals in managing forensic cases, it is essential to detail their specific contributions. Forensic pathologists are responsible for determining the cause and manner of death by analyzing physical evidence, performing autopsies, and interpreting findings in the context of the deceased’s circumstances. Physicians, particularly those with expertise in psychiatry, contribute by assessing the deceased’s medical and mental health history, providing insights into psychiatric conditions that may have influenced their behavior. Mental health providers, such as clinical psychologists or social workers, assist by conducting interviews with families and analyzing interpersonal dynamics, suicide notes, or other behavioral evidence to reconstruct the deceased’s psychological state [44].

The advantages of such a collaborative effort in a forensic context are significant. By combining the expertise of medical and mental health professionals, forensic investigations can achieve a more holistic understanding of the factors contributing to a death. This synergy enhances accuracy in determining the cause and manner of death, supports public health strategies for suicide prevention, and provides more meaningful insights to families seeking closure [44]. However, barriers to effective collaboration remain. Differing professional practices, terminologies, and cultural approaches can hinder communication between forensic pathologists, physicians, and mental health providers. For instance, forensic professionals often prioritize physical evidence, while mental health providers emphasize psychological and social contexts. These differences can lead to fragmented investigations and missed opportunities to fully understand complex cases.

To overcome these barriers, specific recommendations can be proposed [44]. Developing standardized protocols for interdisciplinary collaboration is crucial. Regular joint training programs, case review sessions, and the inclusion of mental health providers in forensic teams could improve communication and mutual understanding. Additionally, creating a framework for sharing information and establishing clear roles for each professional within the team would streamline efforts. By addressing these barriers and enhancing collaboration, forensic investigations can become more effective in uncovering the truth and supporting both legal and public health objectives.

The role of forensic medicine in Italy is deeply intertwined with the country’s epidemiological trends and regional variations in suicide [34,35,36,37]. Forensic pathologists routinely work with public health authorities to analyze suicide patterns, particularly in rural and economically disadvantaged areas. Detailed national data reveal distinct gender- and age-related trends in suicide methods, which guide the forensic investigation process. For example, in Italy, hanging and falling from heights are among the most frequent methods used, with noticeable regional and demographic variations.

## 7. The Suicide Among Cisgender, Transgender, and Intersex People

Suicide is a major public health problem globally, with devastating effects on the individuals involved, their families, and the communities they belong to. Unfortunately, people from the LGBTQ+ community, including cisgender, transgender, and intersex individuals, are disproportionately affected by this tragedy. Statistics and international studies show that suicide and suicide attempt rates in these populations are significantly higher than the general average. This phenomenon is the result of a series of complex factors that include social discrimination, stigma, mental health problems, lack of support, and difficulty in accessing health services, including those dedicated to mental health and psychological support [45]. Studies conducted internationally reveal the seriousness of the situation, particularly among LGBTQ+ youth. It is estimated that between 30% and 40% of LGBTQ+ youth have attempted suicide, with rates of attempts that are 1.5 to 3 times higher than their heterosexual peers. In particular, lesbian, gay, bisexual, and transgender youth are at least four times more likely to attempt suicide than heterosexual youth [45,46,47,48]. These numbers are alarming and suggest an urgent need for interventions to address the root contributing factors of suicide, which are often linked to discrimination and social isolation. Transgender people, in particular, are at increased risk of suicide. Studies in several countries, including Denmark, have found that the risk of suicide among transgender people is 3.5 times higher than in the general population, with an attempt rate that is even 7–8 times higher. These data indicate a significant vulnerability that cannot be ignored. Furthermore, among non-binary populations and other gender identities that differ from the traditional binary, the statistics are equally worrying. In Australia, for example, more than half of trans men reported having attempted suicide at least once in their life, while around a fifth of cisgender men have had similar experiences [45,46,47,48]. Furthermore, 90% of non-binary people reported having suicidal thoughts, highlighting once again the serious vulnerability of these identities compared to the average population. The factors that contribute to these high suicide rates among LGBTQ+ people are multiple and complex. First, social prejudice and stigma are among the main contributing factors. The discrimination, rejection, and ostracism that many LGBTQ+ people face on a daily basis increase their level of stress and isolation, making them more vulnerable to psychiatric disorders and suicidal behaviors [45,46,47,48]. The situation is further complicated for transgender people, who not only face social rejection but also difficulty accessing adequate health services. Despite some legislative progress, many transgender people continue to experience high rates of morbidity and mortality, including deaths by suicide, due to the lack of appropriate health resources, discrimination by professionals in the sector, and the scarcity of accessible psychological support services. It is necessary to promote a culture of inclusiveness and understanding, offer targeted psychological support to LGBTQ+ people, and work to break down the social and institutional barriers that hinder access to adequate treatments. Only through an integrated approach that considers the multiple dimensions of discrimination and marginalization can we hope to achieve these goals.

## 8. Psychological Autopsy: A Tool for Understanding Suicide

In addition to carrying out the investigations described above, the coroner may perform a psychological autopsy. A psychological autopsy is an investigative method used primarily in cases of violent or dubious death, with the aim of understanding the psychological and social circumstances that led to an individual’s death. This practice is distinct from the medico-legal autopsy, which focuses on the physical analysis of the body to determine the cause of death [36]. Psychological autopsy is based on the collection and analysis of information relating to the life of the deceased, including interpersonal relationships, medical history, and circumstances prior to death. For example, in a hypothetical case involving a man found dead in his apartment, the psychological autopsy process begins with extensive interviews with family members and colleagues. These interviews may reveal a recent history of job loss, financial difficulties, or withdrawal from social activities, which provide critical context for understanding the factors contributing to suicide. Simultaneously, clinical records may show a history of untreated depression, and personal writings, including a suicide note, may express feelings of hopelessness and failure [37]. The process involves: Interviews: Extensive interviews are conducted with family members, friends, and acquaintances to gather information about the life and behavior of the deceased; documentation: clinical documents, personal writings, and any other relevant information that can help reconstruct the psychological context of the subject are collected and analyzed [37]. Scene analysis: Analysis of the place where the death occurred is crucial, especially to understand the dynamics that may have contributed to the fatal event, as in the case of suicides or homicides. Psychological autopsy is often used to differentiate between suicide and other contributing factors and can also have a therapeutic value for family members, helping them to accept the loss and understand the motivations of the deceased. Suicide notes are written by the person being investigated by a coroner and are a message left by individuals who intend to commit suicide [38,39]. Research indicates that approximately 25–30% of suicides are accompanied by a note, with some demographics reporting rates as high as 50%. These notes can take many forms, including written messages and audio or video recordings [40]. People may choose to write suicide notes for a variety of reasons, including: to ease grief: to relieve loved ones’ guilt by providing explanations; to create guilt: to intensify the emotional burden on survivors; to explain reasons: to articulate the reasons behind their decision; to send messages: to communicate thoughts and feelings that were difficult to express during life; to provide instructions: to provide directions regarding their remains or their affairs; confession: occasionally, confessing to other crimes, such as murder in murder-suicide cases [41,42,43]. In the context of Italy, psychological autopsy has emerged as a critical tool in forensic investigations, particularly in cases of ambiguous deaths. This method has been employed to reconstruct the psychosocial and medical history of individuals who died by suicide, allowing forensic experts to differentiate between suicide, homicide, and accidental death [34,35]. For instance, in Italian forensic practice, psychological autopsies have been instrumental in understanding the underlying factors contributing to suicides in regions with higher rates, such as Trentino-Alto Adige and Friuli-Venezia Giulia, where socioeconomic isolation plays a significant role [34,35].

### Suicide Notes: Understanding the Final Messages

The medical examiner not only plays a vital role in determining the cause of death in suicide cases, but also contributes to a broader understanding of the social and psychological dynamics that can lead to such acts. Their expertise is essential to ensure that deaths are treated with due care and respect, both medically and legally. Suicide notes are essential to understanding the motivations and emotional states of individuals who take their own lives. They highlight the importance of addressing feelings of guilt and despair, which are prevalent themes in many notes. Further research into the content and context of these notes could improve suicide prevention strategies and support for those in crisis. These manuscripts may also have an explanatory purpose and may have a therapeutic role in helping surviving relatives understand suicide. An understanding of the messages contained in suicide notes could be useful for suicide prevention programs.

## 9. Conclusions: Forensic Medicine’s Contribution to Suicide Understanding and Prevention

From the analysis conducted, it can be clearly seen that the phenomenon of suicide manifests itself significantly differently between men and women, with a much higher incidence in the male population. Italian data show that the male suicide rate is almost four times higher than the female one, and that this disparity has been increasing over the years. Interestingly, although economic and social factors have had a significant impact on the suicide rate, the SARS-CoV-2 pandemic has not produced a dramatic increase as initially expected, suggesting the effectiveness of some support and prevention measures.

Another key aspect that emerged is the correlation between suicide and the presence of comorbidities, especially mental illnesses such as depression and anxiety, which are found to be determining factors in 20% of cases [49,50,51,52]. Furthermore, suicide among men tends to be concentrated in areas with low population density, suggesting that social and economic factors play a crucial role. The lethal methods most chosen by men, such as hanging, and by women, such as falling, are indicative of the seriousness with which these extreme gestures are implemented. The role of forensic medicine is essential not only to determine the cause of death but also to investigate the psychological and social contributing factors that can lead to suicide. The skills of the forensic doctor extend to the analysis of pre-existing medical conditions, psychological autopsy, and the examination of manuscripts left by the deceased, such as suicide notes, which offer important indications on the motivations behind the gesture. The synergy between forensic medicine and psychiatry is crucial for a complete understanding of suicidal dynamics and to develop more effective preventive strategies. Finally, it is clear that greater integration of medical examiners into mental health teams could improve the diagnosis and management of suicidal risks, allowing for timely and targeted intervention. Collaboration between professionals, combined with continuous improvement in training, could not only contribute to the prevention of suicide but also help the families of victims to better understand the reasons for these tragic events, promoting adequate support for their mourning process.

## Figures and Tables

**Table 1:** Summary of Suicide Data in Italy.

Aspect	Details
Total Suicides (2020–2021)	7422 recorded suicides
Gender Distribution (2020–2021)	76.8% male, 23.2% female
Most Common Methods (Male)	Hanging (52.2%), Falling (15.8%), Firearms (13.7%)
Most Common Methods (Female)	Hanging (34.8%), Falling (31.9%), Drowning (8.0%)
Regions with Highest Rates	Northern Italy (particularly North-East regions)
Age Group with Highest Rates (Male)	≥70 years old
Age Group with Highest Rates (Female)	≥70 years old
Impact of COVID-19	Overall reduction in suicides (−2.8% males, −7.7% females); slight increase in elderly males and females ≥ 85 years
Key Associated Comorbidities	Depression, anxiety, physical illnesses (e.g., chronic conditions)
Percentage of Cases with Suicide Notes	25–30% of cases include notes (varies by demographic)

## Data Availability

Not applicable to this article as no datasets were generated.
